# Klotho/FGF23 and Wnt Signaling as Important Players in the Comorbidities Associated with Chronic Kidney Disease

**DOI:** 10.3390/toxins12030185

**Published:** 2020-03-16

**Authors:** Juan Rafael Muñoz-Castañeda, Cristian Rodelo-Haad, Maria Victoria Pendon-Ruiz de Mier, Alejandro Martin-Malo, Rafael Santamaria, Mariano Rodriguez

**Affiliations:** 1 Maimonides Institute for Biomedical Research (IMIBIC), 14005 Cordoba, Spain; juanr.munoz.exts@juntadeandalucia.es (J.R.M.-C.); crisroha@yahoo.com (C.R.-H.); alejandro.martin.sspa@juntadeandalucia.es (A.M.-M.); rsantamariao@gmail.com (R.S.); marianorodriguezportillo@gmail.com (M.R.); 2 School of Medicine, Department of Medicine, University of Cordoba, 14005 Cordoba, Spain; 3 Nephrology Service, Reina Sofia University Hospital, 14005 Cordoba, Spain; 4 Spanish Renal Research Network (REDinREN), Institute of Health Carlos III, 28029 Madrid, Spain

**Keywords:** FGFG23, Klotho, Wnt/β-catenin, CKD, cardiorenal syndrome

## Abstract

Fibroblast Growth Factor 23 (FGF23) and Klotho play an essential role in the regulation of mineral metabolism, and both are altered as a consequence of renal failure. FGF23 increases to augment phosphaturia, which prevents phosphate accumulation at the early stages of chronic kidney disease (CKD). This effect of FGF23 requires the presence of Klotho in the renal tubules. However, Klotho expression is reduced as soon as renal function is starting to fail to generate a state of FGF23 resistance. Changes in these proteins directly affect to other mineral metabolism parameters; they may affect renal function and can produce damage in other organs such as bone, heart, or vessels. Some of the mechanisms responsible for the changes in FGF23 and Klotho levels are related to modifications in the Wnt signaling. This review examines the link between FGF23/Klotho and Wnt/β-catenin in different organs: kidney, heart, and bone. Activation of the canonical Wnt signaling produces changes in FGF23 and Klotho and vice versa; therefore, this pathway emerges as a potential therapeutic target that may help to prevent CKD-associated complications.

## 1. Introduction

Chronic kidney disease (CKD) causes alterations in mineral metabolism, which worsens as the renal disease progresses. It is observed that with only a marginal decrease of glomerular filtration, there is a downregulation of renal α-Klotho (Klotho) [1]. Renal Klotho is the co-receptor of Fibroblast Growth Factor Receptor-1 (FGFR1), the specific receptor of the phosphaturic hormone Fibroblast Growth Factor-23 (FGF23). Thus, FGF23 promotes urinary excretion of phosphate and prevents hyperphosphatemia until the glomerular filtration rate falls below 15–20 mL/min. In addition to α-Klotho, expressed in tubular cell membranes, there are two other types of Klotho: soluble (sKlotho) and secreted Klotho, with additional effects in other organs. Actually, there are studies showing the pleiotropic effects of Klotho in the cardiovascular system [2], bone [3], and even as a tumor suppressor molecule [4,5]. The mechanisms behind this reduction of renal α-Klotho during CKD are unclear, and they are attributed mainly to kidney function deterioration, although Wnt/β-catenin activation has also been suggested as a key factor leading to Klotho reduction [6]. 

FGF23 is a hormone produced mainly in mature osteoblasts and osteocytes, and in addition to its phosphaturic effect, it also inhibits 1,25(OH)_2_D and PTH production [7]. In CKD patients, the concentration of plasma FGF23 increases progressively in part due to kidney resistance to the action of FGF23 generated by the lack of the co-receptor α-Klotho. An experiment in animals demonstrated that the reduction of α-Klotho is precipitated by an excessive tubular load of phosphate [6,8]. In fact, the increase in FGF23 levels is accompanied by a marked decrease in Klotho. Drueke et al. showed a descriptive illustration where it is collected through progressive changes in the parameters of mineral metabolism, and through CKD parameters during renal disease progression [1]. It is interesting to note that in parallel to the decrease of Klotho, and the increase of FGF23, there are also changes in the levels of Wnt inhibitors, such as sclerostin or Dickkopf-related proteins (Dkk1). However, the relationship between the FGF23/Klotho axis and Wnt signaling has not been sufficiently explored. 

Works from different researchers have described an interrelationship between alterations in mineral metabolism and changes in Wnt signaling in the kidney, vessels, heart, bone, and brain, among others. This review will summarize the relationship between Wnt signaling, FGF23, and Klotho expression. 

## 2. The Wnt/β-Catenin Cell Signaling Pathway

**Figure 1:**
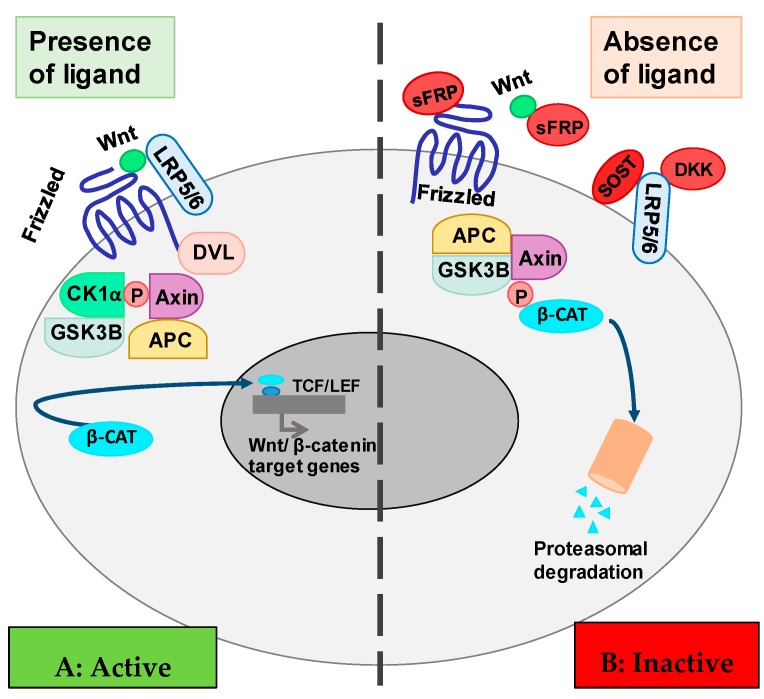
Simplified scheme of the Wnt/β-catenin signaling pathway. (**A**) Wnt ligand interaction with Frizzled protein and LRP5/6. Disheveled (DVL) protein binds the Frizzled receptor and sequester the protein complex CK1a-GSK3-Axin-APC blocking β-catenin phosphorylation and degradation. β-catenin activates TCF/LEF transcription factor in the nucleus. (**B**) Interference of Wnt ligand–Frizzled protein interaction by sFRP, SOST, or DKK1. Disheveled (DVL) protein does not bind to the Frizzled receptor. Protein complex GSK3-Axin-APC phosphorylates β-catenin. Phosphorylated β-catenin is led to proteasomal degradation. Abbreviations: GSK3β: glycogen synthase kinase 3; APC: adenomatous polyposis coli; TCF/LEF: T-cell factor/lymphoid enhancer-binding factor; sFRP: secreted Frizzled-related proteins; SOST: sclerostin; DKK1: Dickkopf-related proteins.

The Wnt pathway is highly conserved in the evolution of animal life. It is classified into several sub-pathways called canonical and non-canonical. The non-canonical Wnt pathways are not dependent on the β-catenin-T-cell factor/lymphoid enhancer-binding factor (TCF/LEF), such as the Wnt/Ca^2+^ pathway and the non-canonical Wnt planar cell polarity [9]. The canonical Wnt pathway involves the nuclear translocation of β-catenin and the activation of the target genes via TCF/LEF transcription factors (Figure 1). The activation of the canonical Wnt pathway requires the binding of the Wnt ligands to the receptors of the Frizzled family, and the interaction with co-receptors lipoprotein-receptor related protein 5 (LRP5) and LRP6. The binding of ligand and receptor stimulates the sequestration of Axin protein by the Disheveled protein, which prevents the formation of the complex necessary for the degradation of β-catenin. In this setting, β-catenin is not phosphorylated, became stabilized, and is translocated into the nucleus. Into the nucleus, it activates the transcription of the Wnt target genes through the interaction with the transcription factors TCF/LEF [10] (Figure 1A).

In the absence of soluble Wnt protein ligands, the protein Axin forms a complex with the proteins adenomatous polyposis coli (APC), Casein kinase 1 isoform α (CK1α), and glycogen synthase kinase 3α (GSK3α). Axin and APC act as scaffold proteins for GSK3β that binds and phosphorylates β-catenin, which is degraded by the proteasome (Figure 1B). 

Some proteins regulate the Wnt/β-catenin pathway by blocking the Wnt ligands and co-receptors. The members of the secreted Frizzled-related protein (sFRP) are proteins that contain a cysteine-rich domain homologous to the putative Wnt-binding site of Frizzled proteins, which inhibit Wnt activation (Figure 1B). Other proteins as sclerostin (the product of the SOST gene) and Dkk1 interact with LRP5/6, and they function as Wnt signaling inhibitors. Sclerostin binds the LRP5/6 receptors, impairs the LRP5/6-Frizzled interaction, and the interaction of the Wnt signaling proteins with the receptors [11,12]. Dkk1 also binds the LRP5/6 receptor and prevents the activation of the Wnt/β-catenin pathway [13] (see Figure 1).

Although there is not much evidence about the direct interaction of FGF23 or Klotho with Wnt elements, it has been shown that the extracellular domain of Klotho binds to multiple Wnt ligands, inhibiting their ability to activate Wnt signaling [14,15]. It is also known that there is a reciprocal relationship between Klotho, FGF23, and Wnt signaling; thus, Wnt signaling dysregulation affects to FGF23 and Klotho levels and vice versa. There is data suggesting potential crosstalk between Wnt/β-catenin signaling and the regulation of Klotho and FGF23. In CKD patients, uremic toxins, phosphate overload, sclerostin, Dkk1, and inflammation may affect Wnt signaling, thus contributing to the progression of CKD-associated comorbidities [16]. This subject will be exposed in the following sections.

## 3. Klotho-FGF23 and Wnt in Chronic Kidney Disease

### 3.1. Regulation of Klotho Expression in the Kidney: The Effect of the Tubular Load of Phosphate

**Figure 2:**
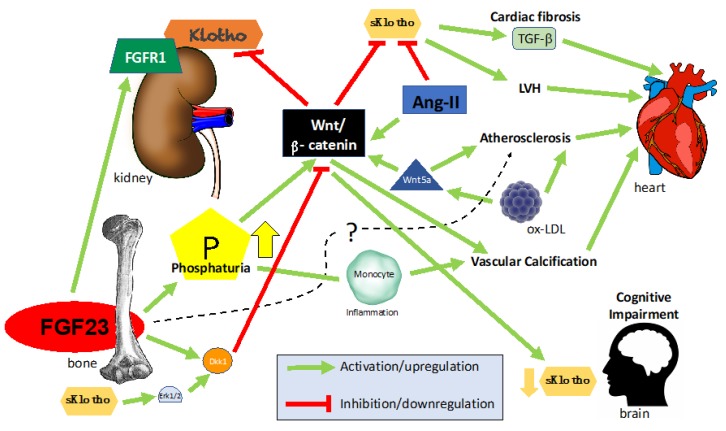
Schematic representation of FGF23/Klotho interactions with the Wnt/β-catenin pathway in the bone, kidney, and heart.

Our group has studied the factors associated with a reduction of renal Klotho expression in rats. Administration of recombinant FGF23 (rFGF23) produced phosphaturia and reduced renal Klotho expression in healthy rats [6]. In 5/6 nephrectomized rats, circulating levels of FGF23 were markedly increased, and Klotho was found to be reduced. In these rats, the administration of anti-FGF23 antibodies further reduced the renal Klotho expression. These results suggest that the increased tubular load of phosphate causes a reduction in Klotho expression. In vitro, HEK-293 cells incubated in high phosphate medium produced nuclear translocation of β-catenin that was followed by a reduction in Klotho expression [6]. We concluded that high phosphate levels decreased renal Klotho expression via activation of the Wnt/β-catenin pathway (Figure 2). The administration of calcitriol to cultured HEK-293 cells prevented Klotho reduction induced by high phosphate.

### 3.2. Klotho, Wnt/β-Catenin, and Renal Damage

Albuminuria downregulates tubular expression of Klotho even in the early stages of CKD [17]. Klotho deficiency induces Wnt activation, which, in turn, is associated with podocyte injury in mice models of diabetic nephropathy and patients with diabetes [18]. Podocyte injury is reduced after the deletion of β-catenin [18]. Although not fully elucidated, Snail-1, a transcription factor induced by Wnt through GSK3β, reduces nephrin expression playing a pivotal role in podocyte injury [19]. The opposite effect is observed with the overexpression of the Wilms Tumor 1 (WT1), which actively suppresses the Wnt pathway through the inhibition of Disheveled protein in podocytes [20]. 

In the mature kidney, Wnt is suppressed, allowing the podocyte to perform its physiological function. Interestingly, Wnt/β-catenin activity may be affected by high glucose; thus, a pathogenic role of Wnt/β-catenin in diabetic nephropathy cannot be ruled out [21]. In this regard, in a mice model of diabetic nephropathy, Klotho seems to protect glomerular and podocyte injury by inhibiting glomerular hypertrophy and reducing albuminuria [22]. Klotho may also reduce proteinuria by blocking the transient receptor potential cation channel (TRPC6) in podocytes [23], and in the heart, Klotho attenuates stress-induced cardiac hypertrophy via inhibition of TRPC6 [24]. 

### 3.3. Klotho, Wnt/β-Catenin, and Polycystic Kidney Disease (PKD)

The Wnt/β-catenin pathway activation is also involved in the development of polycystic kidney disease (ADPKD) [25,26]. These patients show increased plasma levels of FGF23 as compared to GFR-matched CKD patients or healthy volunteers irrespective of the renal function, age, and serum levels of PTH and vitamin D [27]. Furthermore, ADPKD patients exhibit resistance to the renal effect of FGF23 that could be explained by the reduction in Klotho [28]. ADPKD patients show significantly reduced circulating sKlotho, as well as higher FGF23 with a superior FGF23-to-Klotho ratio as compared to healthy volunteers and GFR-matched CKD 1 and 2 patients [28]. Interestingly, sKlotho levels inversely correlate with the total cyst volume and the annual growth of the kidney. 

Cardiovascular disease (CVD) is apparent in ADPKD patients. Indeed, a high incidence of intracranial aneurysms, mitral and aortic valvular prolapse, aortic regurgitation, left ventricular hypertrophy (LVH), and coronary artery disease has been reported, all of which may culminate in heart failure (HF) [29,30,31]. Although some of these manifestations have a genetic background, such as aneurysms, it is also possible that the dysregulation of the FGF23–Klotho complex could play a role in the development of these complications, mainly those that directly affect the heart. However, to our knowledge, no study has described crosstalk between FGF23 increase and Klotho reduction as responsible for ADPKD cardiac and vascular disease.

### 3.4. Consequences of the Close Relationship between Wnt and Klotho

Since Klotho is downregulated in CKD, it is important to delineate this relationship. The fall of renal Klotho is postulated as one of the most important effects of Wnt signaling activation. Therefore, the design of new strategies directed to increase Klotho levels should be considered as a strategy to reduce morbidity and mortality associated with kidney and heart diseases.

sKlotho binds to multiple Wnt ligands suppressing a variety of gene transcription. The upregulation of Klotho halts the activation of Wnt, which reduces the deposition of the extracellular matrix and decreases the transcription of cytokines [32]. The contrary is observed in Klotho heterozygous mutant mice in which Wnt is overexpressed together with an increment of Transforming Growth Factor-β (TGF-β) and collagen type III (Col3); the extracellular matrix deposition and interstitial fibrosis are remarkable as compared to the wild type. Hence, in vivo models have demonstrated that sKlotho attenuates renal fibrosis by halting Wnt signaling [33].

### 3.5. Kidney Fibrosis

Recently, the Wnt pathway in the kidney has gained attention because of its association with renal fibrosis. In the kidney, pericytes are recognized as collagen-producing cells [34]. Once a kidney injury is established, pericytes migrate to the interstitial space where they differentiate into scar-forming myofibroblasts [34]. In this context, the Wnt pathway is markedly activated in pericytes at the time they differentiate into myofibroblasts, which may cause fibrosis of the kidney interstitium [35]. 

### 3.6. Cardiorenal Syndrome

Cardiorenal syndromes have been defined as “disorders of the heart and kidneys whereby acute or chronic dysfunction in one organ may induce acute or chronic dysfunction of the other [36]. There are five subcategories of cardiorenal syndrome based on the primary damaged organ (heart or kidney) and the time course of progression (acute or chronic) [36]. Type 2 cardiorenal syndrome (CRS2) is where HF causes renal dysfunction, and type 4 CRS (CRS4) is where advanced CKD promotes heart dysfunction.

With respect to CRS4, clinical reports have substantiated a relationship between heart and kidney disease [37]. It appears that Klotho deficiency may contribute to the generation of cardiac hypertrophy observed in patients CKD stages G3a–b and G4; however, publications on this issue are limited [38].

In the case of CRS2, a study on a mice model of HF has shed light on the potential mechanisms connecting cardiac and renal dysfunction [39]. In these mice, the constriction of the aorta induced LVH and HF. The increased cardiac remodeling was associated with a significant reduction of Klotho and activation of the Wnt/β-catenin and renin–angiotensin system (RAS). The Wnt/β-catenin activation mediates the injury in both organs, heart, and kidney. Once HF is established, the renal expression of podocalyxin was reduced while fibronectin and Snail1 expression increased, resulting in kidney interstitial fibrosis and albuminuria [39].

Furthermore, different cardiac Wnt ligands increased together with β-catenin, angiotensin-converting enzyme (ACE), renin, and angiotensin I (AT1) expression. Perhaps, the most important finding of this research is that the inhibition of the cardiac-secreted Wnt/β-catenin/RAS axis prevented kidney injury by a downregulation of the expression of fibronectin, Snail1, ACE, renin, and AT1 in the kidney with a consequent reduction of kidney interstitial fibrosis. All the cardiac lesions worsened in association with renal-dependent Klotho depletion. The presence of sKlotho partially inhibited Wnt/β-catenin signaling, which in turn promoted the downregulation of cardiac fibronectin and α-smooth muscle actin. Therefore, the activity of Wnt/β-catenin in the heart is accompanied by kidney injury. Concomitantly, Klotho deficiency resulting from kidney failure worsens cardiac remodeling and function. This finding is not surprising since Klotho deficiency, high serum phosphate, and elevated FGF23 have been demonstrated to modulate cardiac remodeling [40] (Figure 2). 

In CKD patients, more information on the relationship between serum Klotho and FGF23 levels and the values of the Wnt ligands, such as Wnt1, Wnt3a, or Wnt10b, could be useful to assess the comorbidities dependent on the Wnt signaling system, such as renal and cardiac fibrosis. 

## 4. FGF23/Klotho/Wnt in Cardiovascular Disease (CVD)

The involvement of Wnt signaling activation in the pathogenesis of CVD has been widely documented. In addition to the cardiovascular development during embryogenesis, Wnt signaling participates in many cardiac and vascular pathological processes such as RAS alterations, cardiac fibrosis and hypertrophy, atherosclerosis, vascular calcification, endothelial dysfunction, myocardial infarction, or arrhythmias [41] (Figure 2).

### 4.1. Klotho, Wnt/β-Catenin, and RAS

Another point of interest is the association between Wnt/β-catenin signaling and the Pro-Renin Receptor (PRR), a component of the RAS, and critical regulator of blood pressure [42]. In the kidney, PRR is involved in nephron formation, podocytes instability, blood pressure regulation, and sodium transport [42,43]. PRR increases as CKD progress; however, the mechanisms leading are not fully understood. A recent study has demonstrated that Wnt/β-catenin stimulates PRR mRNA expression of in a dose-dependent manner [42]. Similarly, PRR overexpression triggers Wnt gene transcription, perpetuating a cycle that exacerbates kidney fibrosis and a decline of renal function. Multiple RAS genes are direct targets of Wnt/β-catenin signaling [44]. 

However, to date, there is no evidence showing the relationship between Klotho reduction and PRR expression. Given that CKD and Wnt/β-catenin signaling regulate PRR expression, and FGF23 enhances sodium reabsorption through sodium/chloride cotransporter in the distal tubule [45,46], it can be hypothesized that increased FGF23, Wnt activation, and the downregulation of Klotho in CKD may promote volume overload and an elevation in blood pressure, two well-known risk factors for heart failure. Nevertheless, this remains speculative and requires further investigation. Therefore, FGF23, Klotho, and the Wnt pathway may have relevance in the control of blood pressure and RAS. 

### 4.2. FGF23/Klotho and Cardiac Hypertrophy

An increase in the FGF23/Klotho ratio is present since the early stages of CKD, and it is associated with CVD, especially with LVH [47] and vascular calcification [48,49]. Studies by Myles Wolf’s group demonstrated that high levels of FGF23 caused LVH [50]. It is reasonable to speculate that FGF23 through a Wnt signaling activation might be a cause of LVH. In the experimental setting, and with respect to left ventricular remodeling, it is observed that Wnt signaling inhibition improves cardiac function; sFRP or Disheveled, both Wnt inhibitors, attenuate left ventricular remodeling [51]. 

Experimental studies have shown a cardioprotective effect of Klotho, although the mechanisms are unknown. Yu et al. [52] observed that Klotho reduced the Angiotensin II-induced hypertrophic growth of neonatal cardiomyocytes. In these cells, Angiotensin-II promoted Wnt/β-catenin activation while Klotho decreased it. Klotho administration also suppressed the expression of Angiotensin-II receptor type I showing that Klotho might be considered as an antihypertrophic factor useful in heart diseases. 

A recent study has shown in hemodialysis patients that higher serum FGF23 and lower sKlotho and sclerostin (an endogenous Wnt inhibitor) levels were associated with chronic inflammation, malnutrition, secondary hyperparathyroidism, and may be considered as predictors of cardiovascular complications, such as LVH, acute coronary syndrome, or rhythm disturbances [53]. 

### 4.3. FGF23–Klotho–Wnt and Cardiac Fibrosis

During cardiac fibrosis some of the evidences showing a crosstalk of FGF23 and Klotho with Wnt signaling are described. Cardiac fibrosis is characterized as an excessive accumulation of fibroblasts, myofibroblasts, and extracellular matrix proteins in the myocardium [54]. Human hearts with severe epicardial fibrosis show increased activation of β-catenin and TCF/LEF [55]. Additionally, TGF-β is a key profibrotic cytokine in the development of cardiac fibrogenesis. It has been proposed that TGF-β activates Wnt/β-catenin signaling through the production of Wnt proteins, and by direct deactivation of GSK3β. Activated Wnt/β-catenin, in turn, stabilizes the TGF-β/Smad response. It appears that the co-activation of these two pathways is required to trigger the effective fibrotic response [56]. Akhmetshina et al. showed that canonical Wnt signaling activation is required for TGF-β-mediated fibrosis [57]. Recently, Liu Q et al. showed through in vitro studies that the loss of endogenous cardiac Klotho in CKD patients, specifically in cardiomyocytes, intensifies TGF-β1 signaling, which enables more vigorous cardiac fibrosis through upregulation of Wnt signaling. Moreover, the upregulation of endogenous Klotho inhibited Wnt/β-catenin signaling [58], a desirable strategy for the prevention and treatment of cardiac fibrosis in CKD patients. 

Other authors have shown that secreted Klotho can inhibit TGF-β1 signaling through its interaction with TGF-β1 cell-surface receptors [59]. 

With respect to FGF23, Hao et al. observed that in cultured adult mouse cardiac fibroblasts, rFGF23 increased active β-catenin, procollagen I, and procollagen III expression [60]. Schumacher et al. showed that FGF23 increased the expression of Collagen 1, MMP8, and fibronectin in cardiac fibroblasts; in addition, they showed that high levels of FGF23 increased the expression of TGF-β1 in M2 polarized macrophages [61]. So, FGF23 might be involved also in cardiac fibrosis generation.

These studies reveal a close association between Klotho, TGF-β, and Wnt signaling activation in the generation of cardiac fibrosis. The evaluation of these parameters in the context of clinical studies will determine if modulation of Wnt signaling could be a potential therapeutic target. 

### 4.4. FGF23/Klotho and Atherosclerosis

Vascular endothelial dysfunction is one of the first events in the atherosclerotic process. The endothelial injury allows monocyte adhesion with subsequent infiltration into the subintimal space. Subsequently, these monocytes are differentiated into macrophages, beginning an inflammatory process with the release of proinflammatory cytokines and nuclear translocation of NF-kB. This inflammatory process produces changes in vascular smooth muscle cells (VSMC) from contractile to a synthetic phenotype with a higher capability to migrate from the media to the intima layer in arteries. In this space, both macrophages and VSMC accumulate lipids resulting in the formation of an atherosclerotic plaque with a fibrous cap on the luminal side of the vessel [62]. 

The atherosclerotic process is also associated with Wnt signaling activation. There is a positive correlation between the severity of the atherosclerotic lesion and serum Wnt5a levels. Moreover, Wnt5a staining has been detected in intimal areas of macrophage accumulation in atherosclerotic lesions of apolipoprotein-deficient mice, and human endarterectomy samples. Christman et al. showed that oxidized LDL induced Wnt5a expression, a potential mechanism to activate Wnt signaling (Figure 2) [63]. Other authors have shown that elevated concentrations of oxidized LDL induce a decrease in renal Klotho expression [64].

Similarly, in human umbilical vein endothelial cell (HUVEC), recombinant Klotho supplementation can attenuate oxidized-LDL-induced oxidative stress through upregulating oxidative scavengers (SOD and NO) [65]. Certainly, more studies are necessary to confirm the potential interaction between oxidized LDL, Klotho, Wnt, and atherosclerosis progression.

In a recent publication, we have reported a significant association between FGF23 levels and carotid intimal media thickness. In 939 subjects with coronary heart disease without CKD enrolled in the CORDIOPREV study, we found that FGF23 was independently associated with intima-media thickness of both common carotid arteries [66].

Chen et al. observed that in hemodialysis patients, sclerostin was also positively associated with carotid intima-media thickness, and patients with low baseline serum sclerostin displayed a better survival. Interestingly, in this study, the authors found a negative association of sclerostin with sKlotho [67]. Although in these patients, low sKlotho levels are caused by the advanced state of CKD, it is unknown if low sKlotho levels also cause high levels of sclerostin. At the moment, it is unknown whether FGF23 through the Wnt signaling might promote the atherosclerotic process. 

### 4.5. FGF23/Klotho and Vascular Calcification

Vascular calcifications are common in patients with advanced CKD, and at present, it is responsible for the high CVD-related mortality [68]. Vascular calcification is the final consequence of a process where VSMC are transdifferentiated into osteoblast-like cells [69].

Patients with end-stages CKD have an important disbalance of mineral metabolism with high levels of serum phosphate, which have been associated in vivo, and in vitro, with the generation of vascular calcification [70]. Different authors and our group have shown that high phosphate levels can activate Wnt signaling in VSMC [71,72]. Interestingly other studies have shown that Klotho supplementation may prevent VSMC calcification through inhibition of the Wnt/β-catenin pathway [73].

We have investigated the relationship between vascular calcification, inflammation, and Wnt signaling. The administration of lipopolysaccharide (LPS) to healthy rats produced inflammation and a parallel increase in serum FGF23 levels and a reduction in renal Klotho expression. Subsequently, ex vivo experiments using slices of kidney tissue showed that LPS and also high phosphate-induced nuclear translocation of β-catenin and p65-NF-kB, with a decrease in Klotho. Inhibition of both inflammation and Wnt signaling activation decreased FGF23 levels and increased renal Klotho [74] (Figure 2). These results support the close relationship between inflammation, impairment in phosphate regulation, calcification, Klotho, and Wnt signaling. 

The potential direct effect of FGF23 on VSMC to promote or inhibit calcification remains controversial. Some authors have found that FGF23 directly increases VSMC calcifications, while other authors emphasized that FGF23 is not involved in this process [75,76,77]. Similarly, it is unknown if FGF23 might or might not promote changes on the VSMC phenotype, with loss of vascular function, atherosclerosis, or even arterial stiffness [78].

## 5. FGF23-Klotho and Wnt in Bone

Historically, the participation of the Wnt/β-catenin pathway in bone disorders has been widely documented. SOST gene produces sclerostin that modulates the Wnt activity. Without sclerostin, Wnt activity is unrestricted, producing increased bone mineral density with hyperostosis. Thus, the canonical Wnt pathway is critical in bone formation, and its modulation could be a target in the treatment of bone disorders.

In relation to CKD, two inhibitors of the canonical Wnt pathway have been investigated: Dkk1 and sclerostin [79]. Paradoxically, despite both molecules inhibiting the Wnt ligand–LRP5/6–Frizzled interaction, the downstream responses are different, illustrating the complexity of this pathway. In CKD patients, the correlation of serum Dkk1 with mineral and bone parameters is nonexistent in most studies [80,81], suggesting that Dkk1 might have a weak relation with renal osteodystrophy. Nevertheless, the serum sclerostin levels increase early in CKD before renal osteodystrophy is established. Sclerostin is produced and secreted by osteocytes, suggesting an essential role in the relationship between bone, kidney, and Wnt in CKD patients [82]. Serum sclerostin levels are higher in males than females, and the levels do not correlate with age. In CKD patients, plasma sclerostin increases progressively as the glomerular filtration rate declines, and it correlates with serum phosphate [83]. The cause of increased plasma levels of sclerostin in CKD patients is unknown. Osteocytes produce sclerostin, and VSMC transdifferentiated into osteoblast in calcified vessels. Likewise, there is limited information about the relationship between sclerostin levels and bone in CKD. Paradoxically, there is a positive association between serum sclerostin levels and bone mineral density in hemodialysis patients [84]. Additionally, the administration of neutralizing antibodies against sclerostin in a murine model of CKD resulted in beneficial only in low PTH conditions [85].

There are many questions in relation to sclerostin and CKD that remain to be answered. It is unknown if high levels of sclerostin protect against vascular calcification where Wnt/β-catenin promotes osteogenic transdifferentiation of VSMC; it is also unclear to what extent high sclerostin affects bone turnover and renal osteodystrophy. The relationship between sclerostin and other mineral metabolism parameters, such as PTH, FGF23, vitamin D, or Klotho, is also unclear. Perhaps more studies are necessary to characterize the effects of Wnt activity on bone metabolism in CKD.

With respect to FGF23, Carrillo et al. identified that FGF23 directly inhibits Wnt signaling through the increase of Dkk1 levels. This action occurs in bone with the participation of soluble Klotho (sKlotho) [86]. This work provides evidence of the autocrine effects of FGF23, which could contribute to the generation of renal osteodystrophy (Figure 2). These results would be aligned with those indicating that an increase of sclerostin would contribute to the inhibition of osteogenesis. A recent study has shown a positive correlation between FGF23 and sclerostin levels in patients with rheumatic arthritis, suggesting a link between FGF23, reduced Wnt activity, and bone demineralization in these patients [87]. Evenepoel et al. found that sclerostin but not Dkk1 participate in alterations of mineral metabolism related to CKD [79,81].

In vitro studies have shown an interaction between FGF23, Klotho, and Wnt signaling in bone cells. The presence of Klotho in osteocytes and osteoblasts [88], suggests that the bone is another target organ for FGF23. Several studies indicate that Klotho is a negative modulator of bone formation [3]. The mechanisms are not clear, but it is speculated that Klotho allows FGF23 to enhance Dkk1 expression resulting in inhibition of Wnt signaling and osteogenesis. This hypothesis is supported by previous observations showing that Wnt activity is increased in Klotho knockout mice [89]. Ma et al. observed that in UMR-106, a bone cell line, the addition of β-glycerophosphate increased the expression of Wnt target genes; the co-administration of β-glycerophosphate and sKlotho led to a decrease in FGF23 levels and a reduction in Wnt activation, suggesting that sKlotho could modulate osteogenesis and FGF23 production [90]. In this line, other authors have observed that secreted Klotho, through the inhibition of FGFR1 and ERK phosphorylation, can delay human mesenchymal stem cell differentiation into osteoblasts [91,92] (Figure 2).

## 6. Wnt and the Central Nervous System

FGF23, FGF receptors (FGFR), and the co-receptor Klotho are also expressed in the central nervous system. The biological relevance of the FGF23/Klotho system in the brain is uncertain, but there is some evidence that FGF23 directly acts on hippocampal neurons reducing memory functions and learning capacity in CKD patients [93,94]. Low serum Klotho levels have been reported to be associated with cognitive impairment [95]; however, the mechanisms are unknown. Given that Klotho is an antagonist of endogenous Wnt/β-catenin activity [32], it is reasonable to speculate that if Klotho reduces Wnt activity, upregulation of Wnt could be associated with cognitive impairment. Klotho-deficient mouse models rapidly develop cognitive impairment and show some evidence of neurodegeneration. In humans, there are reports showing a correlation between Klotho deficiency with dementia and Alzheimer’s [96]. Different explanations may support this association. First, both FGF23 increase and Klotho deficiency are associated with a vascular disease, which may cause cognitive deterioration because based on vascular dysfunction. Second, Vitamin D deficiency is highly prevalent in CKD patients, and alterations mainly mediate in the FGF23/Klotho axis. Vitamin D deficiency has also been related to cognitive decline in older adults. Third, Klotho plays a critical role in life-extension by regulating telomere length and telomerase activity. Both Klotho and telomeres regulate the stem cell aging process through Wnt signaling [97] (Figure 2). Klotho deficiency results in continuous activation of Wnt signaling and senescence of stem cells [98]. Long-lasting activation of Wnt signaling may cause rapid exhaustion and depletion of neural stem cells. Since stem cell dysfunction limits tissue regeneration and potentially affects aging processes, the ability of secreted Klotho protein to inhibit Wnt signaling may reduce aging-like phenotypes in Klotho-deficient mice. Li et al. have shown that Klotho improves memory performance but disturbs some aspects of social behavior [99]. This has been proven in in vivo experiments, in which the addition of only the secreted Klotho protein improves the learning and memory capabilities of old animals [100]. Klotho is also being considered a new therapeutic target of neurodegenerative diseases [101]. Since CKD patients have an increase of FGF23 and a reduction of vitamin D and Klotho levels, it could be hypothesized that CKD patients may also show a decrease in the production of cerebral Klotho, which would upregulate Wnt signaling and produce the cognitive dysfunction frequently observed in these patients. 

## 7. FGF23/Klotho and Wnt in Other Organs

In the lungs, low Klotho may contribute to the development of idiopathic pulmonary fibrosis [102]. The co-administration of Klotho with rFGF23 reduced fibrosis and inflammation through the inhibition of the TGF-β signaling and the decrease in SMAD3 phosphorylation. Klotho relevance on pulmonary disease is reinforced by recent evidence suggesting that less circulating Klotho correlates negatively with lung function parameters, such as the forced vital capacity (FVC), the forced expiratory volume in 1 s (FEV1), and the diffusing capacity of the lung for carbon monoxide (DL_CO_) [102,103]. 

Chronic obstructive pulmonary disease (COPD) is associated with the downregulation of Klotho expression in the airways and an increase in circulating FGF23 levels [104]. Oxidative stress produced by cigarette smoking may be responsible for Klotho deficiency in such a population. Moreover, COPD patients also show elevated inflammatory parameters that may increase FGF23 production, which in turn induce the expression of locally secreted IL-1β in bronchial epithelial cells [105]. Interestingly, the instillation of sKlotho protects bronchial epithelial cells from the pro-inflammatory actions associated with cigarette smoke and FGF23 [105]. Nevertheless, the precise mechanisms whereby pulmonary Klotho expression is downregulated remain undefined. Wnt/β-catenin signaling has recently gained relevance after the demonstration of enhanced noncanonical Wnt-5a activation in human fibroblasts from COPD patients, causing enlargement and destruction of alveolar space and contributing to emphysema development [106]. The inhibition of the Wnt-5a pathways in the lung helps to recover alveolar cell functions, perhaps through the regulation of TGF-β activity by Wnt-5a [107]. Upregulation of Wnt signaling is also associated with an increment in pulmonary vascular resistance, leading to the development of pulmonary arterial hypertension [41]; upregulation of the Wnt/β-catenin pathway has been associated with the proliferation of pulmonary artery smooth muscle cells, pulmonary artery resistance, and heart failure. 

Our opinion is that the evidence is limited, and further investigation to define whether deficiency of lung Klotho and Wnt/β-catenin signaling plays a role in pulmonary fibrosis and emphysema is required. 

Concerning the liver, a specific effect of α-Klotho in the liver is only partially defined. To date, there is no evidence of a detrimental effect of the FGF23/Klotho complex in the liver. In fact, FGF23 promotes hepatocytes proliferation and cytokine production [94,108], despite the lack of expression of Klotho in hepatocytes [94]. Thus, FGF23 action on the liver is Klotho independent, and it is mediated by FGFR4 [46,108]. The Wnt/β-catenin, together with different FGFs, are pivotal in hepatobiliary development; early in embryonal development, β-catenin warrants hepatoblast proliferation and conversion into hepatocytes [109].

The liver is tightly associated with multiple endocrine functions, such as energy homeostasis. In this line, it seems that sKlotho improves insulin sensitivity and insulin release, and it reduces lipid accumulation in the liver [110]. Reciprocally, β-Klotho likely preserves liver integrity by serving as co-receptor for endocrine FGF21, a liver-derived hormone and member of the FGF family that promotes thermogenesis and glucose uptake in adipose tissue [111]. Chronic liver injuries frequently evolve liver fibrosis with a loss of function. There is data suggesting that the Wnt/β-catenin pathway is the main regulator of liver fibrosis. Both Wnt-5a and TGF-β are related to myofibroblast proliferation, collagen deposition, and fibrosis of the liver [41,112]. Nonetheless, dysregulation of Klotho has not been identified as responsible for liver fibrosis. 

In summary, a consequence of the deterioration of kidney function is the modification of regulatory systems in an attempt to restore homeostasis. However, the “price to pay” is that these adaptations may disrupt the physiology, and comorbidities may become apparent. The Wnt/β-catenin cell signaling pathway has gained attention, given the demonstrated role in the development of different CKD-associated comorbidities. Phosphate overload downregulates renal Klotho expression through the activation of the Wnt/β-catenin signaling pathway, thus contributing to the development of vascular calcification and the alteration of the regulation of mineral metabolism. 

The activation of Wnt/β-catenin has other consequences; it promotes tissue fibrosis in both kidney and heart and, more importantly, the upregulation of Wnt/β-catenin may facilitate the crosstalk between the heart and kidney playing a critical role in the development of the cardiorenal syndrome. It is important to note that activation of FGF23/Klotho/Wnt signaling correlates with the severity of atherosclerotic plaques, carotid intimal media thickness, and VSMC calcification. As such, the current evidence suggests that Wnt/β-catenin activation plays an essential role in CKD progression and cardiovascular disease. Therefore, the Wnt/β-catenin pathway may deserve future evaluation as a potential therapeutic target aiming to reduce the prevalence of CKD-associated comorbidities.

## Abbreviations

FGF23	Fibroblast Growth Factor 23
CKD	Chronic kidney disease
FGFR1	Fibroblast Growth Factor Receptor-1
1,25(OH)_2_D	1,25 hydroxyvitamin D
PTH	Parathyroid Hormone
Dkk1	Dickkopf-related protein-1
TCF/LEF	T-cell factor/lymphoid enhancer-binding factor
LRP5	Lipoprotein-receptor related protein 5
APC	Adenomatous Polyposis Coli
CK1α	Casein kinase 1 isoform α
GSK3β	Glycogen Synthase Kinase 3β
sFRP	Secreted Frizzled-Related Protein
SOST	Sclerostin
DVL	Disheveled
TRPC6	Transient Receptor Potential Cation Channel
CRS	Cardiorenal Syndrome
PKD	Polycystic kidney disease
ADPKD	Autosomal Polycystic kidney disease
CVD	Cardiovascular disease
LVH	Left Ventricular Hypertrophy
HF	Heart Failure
ACE	Angiotensin-Converting Enzyme
AT1	Angiotensin I
PRR	Pro-Renin Receptor
RAS	Renin-angiotensin system
VSMC	Vascular Smooth Muscle Cells
HUVEC	Human Umbilical Vein Endothelial Cell
CIMT	Carotid Intima-Media Thickness
LPS	Lipopolysaccharide
COPD	Chronic Obstructive Pulmonary Disease
